# *VEGFA* rs3025039 and biliary atresia susceptibility in Chinese population: a systematic review and meta-analysis

**DOI:** 10.1136/wjps-2021-000344

**Published:** 2022-01-06

**Authors:** Shiwei He, Yifan Yang, Lingdu Meng, Gong Chen, Yanlei Huang, Zhen Shen, Rui Dong, Shan Zheng

**Affiliations:** Department of Pediatric Surgery, Children’s Hospital of Fudan University, Shanghai Key Laboratory of Birth Defect, Shanghai, China

**Keywords:** genetics, pediatrics, evidence-based medicine, epidemiology, gastroenterology

## Abstract

**Background:**

Previous studies have suggested an association between vascular endothelial growth factor A (*VEGFA*) rs3025039 polymorphism and biliary atresia (BA). However, this conclusion is controversial and there is no published pooled evidence of this association.

**Methods:**

This study was conducted and reported according to the Preferred Reporting Items for Systematic Reviews and Meta-Analyses. The protocol was registered with PROSPERO (International Prospective Register of Systematic Reviews). A thorough search was performed on databases including PubMed, Embase, and Chinese Biomedical Database up to August 2020. This study included 846 cases of BA and 2821 controls concerning *VEGFA* rs3025039 polymorphism. We selected relevant studies based on the following inclusion criteria: (1) the study design was case–control and cohort and (2) the patients carried standard clinical diagnoses of BA, etc. The exclusion criteria were as follows: (1) patients with other related diseases, (2) lack of requisite information and (3) duplicate data. The OR (odd ratio) and the corresponding 95% CI (confidence interval) were calculated to estimate the association.

**Results:**

This study on *VEGFA* rs3025039 polymorphism in the Chinese population included 846 cases and 2821 controls. The results showed that there was no significant association between rs3025039 and susceptibility to BA under four genetic models. The results of the subgroup analysis were similar to the overall results.

**Conclusions:**

This meta-analysis shows that rs3025039 was not associated with susceptibility to BA in the Chinese population. Further validation may entail additional research.

**PROSPERO registration number:**

CRD42020203812.

**Key messages**

**What is already known about this subject?**

- The etiology of biliary atresia (BA) is still not well understood.
- Previous studies supported that vascular endothelial growth factor A (*VEGFA*) plays an important role in the pathogenesis of BA.
- The association between the single nucleotide polymorphism(SNP) (rs3025039) in the *VEGFA* gene and the risk of BA is still controversial due to inconsistency among the previous studies.

**What are the new findings?**

- *VEGFA* rs3025039 polymorphism may not be associated with an elevated risk of BA in Chinese.
- The association between rs3025039 and risk of BA was not significant in southern Chinese.
- This meta-analysis did not demonstrate a definite association between rs3025039 and BA in northwestern Chinese because only one study was included in this subgroup.

**How might it impact on clinical practice in the foreseeable future?**

- Additional research is needed to obtain a definite conclusion of this association.
- Future studies are recommended to identify other possible genetic markers.

## Introduction

Biliary atresia (BA) is a type of progressive obliterative disorder in neonates that interferes with the function and anatomy of the intrahepatic and extrahepatic bile ducts.^1 2^ This destructive inflammatory obliterative cholangiopathy frequently leads to hepatic fibrosis and end-stage liver disease. If untreated, BA with progressive liver cirrhosis is uniformly fatal.^3^ The clear etiology of this disorder is not well understood. Genetic and immunological factors, infections, and other environmental factors might lead to BA, suggesting that it has a complex etiology.^3 4^ Hereditary factors participate in the pathogenesis of BA. Multiple single-nucleotide polymorphisms on the genes, including *ADD3*, *XPNPEP1*, *VEGFA*, and *EFEMP1*, are associated with risk of BA.^4^

The human *VEGFA* gene is one of the members of the VEGF (vascular endothelial growth factor) family located on chromosome 6p21.3. This gene comprises a type of heparin-binding protein that is in the form of a disulfide-linked homodimer. *VEGFA* participates in various developmental processes, including endothelial cell proliferation, cell migration, and apoptosis.^5–7^ It may also participate in the pathogenesis of BA because it can function as a proinflammatory cytokine.^8^ Polymorphisms of this functional gene may affect expression regulation, leading to various incidences and severities of disease.^9^ Thus, clarifying the effects of alterations within the *VEGFA* gene may provide markers for diagnosis and treatment to reverse progression of BA.

Previous studies have explored the correlation between rs3025039 within the *VEGFA* gene and susceptibility to BA; however, the results remain controversial due to the lack of consistency among the studies. In China, rs3025039 has been found to be associated with susceptibility to BA.^10 11^ Interestingly, a recent publication reported no significant correlation between rs3025039 and BA in the southern Chinese population.^12^

This discrepancy needs further verification by increasing the sample size. In the mean time, a meta-analysis is a suitable method to summarize previous genetic association studies (GAS) and to draw relatively reliable conclusions.^13 14^ Therefore, the present meta-analysis may provide evidence regarding the association of *VEGFA* rs3025039 polymorphism with susceptibility to BA.

## Methods

This study protocol was registered with an international registration platform of systematic review, PROSPERO (International Prospective Register of Systematic Reviews). We conducted and reported the study according to the Preferred Reporting Items for Systematic Reviews and Meta-Analyses (PRISMA) statement^15^ (see online supplemental table S1).

10.1136/wjps-2021-000344.supp2 Supplementary data

### Patient and public involvement

Patients and the public were not involved in this study.

### Data sources and searches

PubMed, Embase, and the Chinese Biomedical Database (CBD) were searched from inception until August 17, 2020 (see online supplemental material for full details on search strategy). We also considered references in the included studies and related reviews. We did not impose limitations on the language of papers, time period of follow-up, and published state. We reran the same searches before the final analyses and retrieved additional studies for inclusion.

10.1136/wjps-2021-000344.supp1 Supplementary data

### Eligibility criteria

We established the inclusion and exclusion criteria based on discussion studies. We selected relevant studies based on the following inclusion criteria: (1) the study design was case–control and cohort; (2) the patients carried a standard clinical diagnosis of BA; (3) the studies explored the target association; and (4) the authors presented enough data on genotype distribution. The criteria used to exclude studies were as follows: (1) patients with other related diseases; (2) lack of requisite information; and (3) duplicate data.

### Study selection

At the first stage, duplicates from three electronic databases were screened and removed independently by three reviewers (SH, YY, and LM). At the second stage, the title and abstract of each of the remaining studies were reviewed independently by the same reviewers (SH, YY, and LM) to select eligible studies. At the final stage, the same reviewers (SH, YY, and LM) independently retrieved and assessed the potentially eligible full text of the remaining publications. Any discrepancy with regard to eligibility of articles was discussed among the three reviewers in consultation with a third reviewer (RD or SZ).

### Data extraction

All data were recorded independently by three reviewers (SH, YY, and LM) in accordance with a record form, with regard to (1) study characteristics (author information, publication time, sample size, country, and ethnic origin); and (2) genotype data (number with different genotypes, minor allele frequency, results of the Hardy-Weinberg equilibrium (HWE) of the control group, and genotyping methods). All related data were found in the original studies so we did not need to contact the study authors to request for missing data.

### Quality score assessment

Three reviewers (SH, YY, and LM) independently appraised the quality of the GAS using a checklist revised from previous studies,^16 17^ which was done on the basis of genetic factors and epidemiological requirements. The checklist covered essential items aimed at the quality of the GAS, including representativeness and ascertainment of study subjects, genotyping, HWE, and association analysis.^16 17^ The total score ranged from 0 (worst) to 13 (best). Detailed information is shown in online supplemental table S2.

### Statistical analysis

In a GAS, research candidates should be categorized into three groups (BB, Bb, and bb) and usually B is used as the susceptibility allele.^14 18 19^ Previous studies have suggested that the C allele increased BA susceptibility^11^; therefore, we estimated the association between rs3025039 and susceptibility to BA using four different genetic models, namely a per-allele model (C vs T), a homozygous model (CC vs TT), a dominant model (CC+CT vs TT), and a recessive model (CC vs CT+TT).^19^ We measured the effects using OR (odd ratio) and 95% CI (confidence interval) using a fixed-effect or a random-effect model.^20^ We assessed heterogeneity using the Cochrane Q statistic and the inconsistency index (I^2^).^21^ I^2^>50% or p value <0.1 indicated substantial heterogeneity. We conducted subgroup analyses according to the several study subject areas. We performed a sensitivity analysis by excluding every publication individually to evaluate the reliability and stability of the overall OR. We appraised publication bias using Egger’s test and Begg’s test^22^ and visual inspection of funnel plots.^23^ The meta package (V.4.9.7) in R software (V.3.6.1) was used to complete all analyses. In addition to heterogeneity, p<0.05 (two-tailed) indicated significance.

## Results

### Search findings

**Figure 1:**
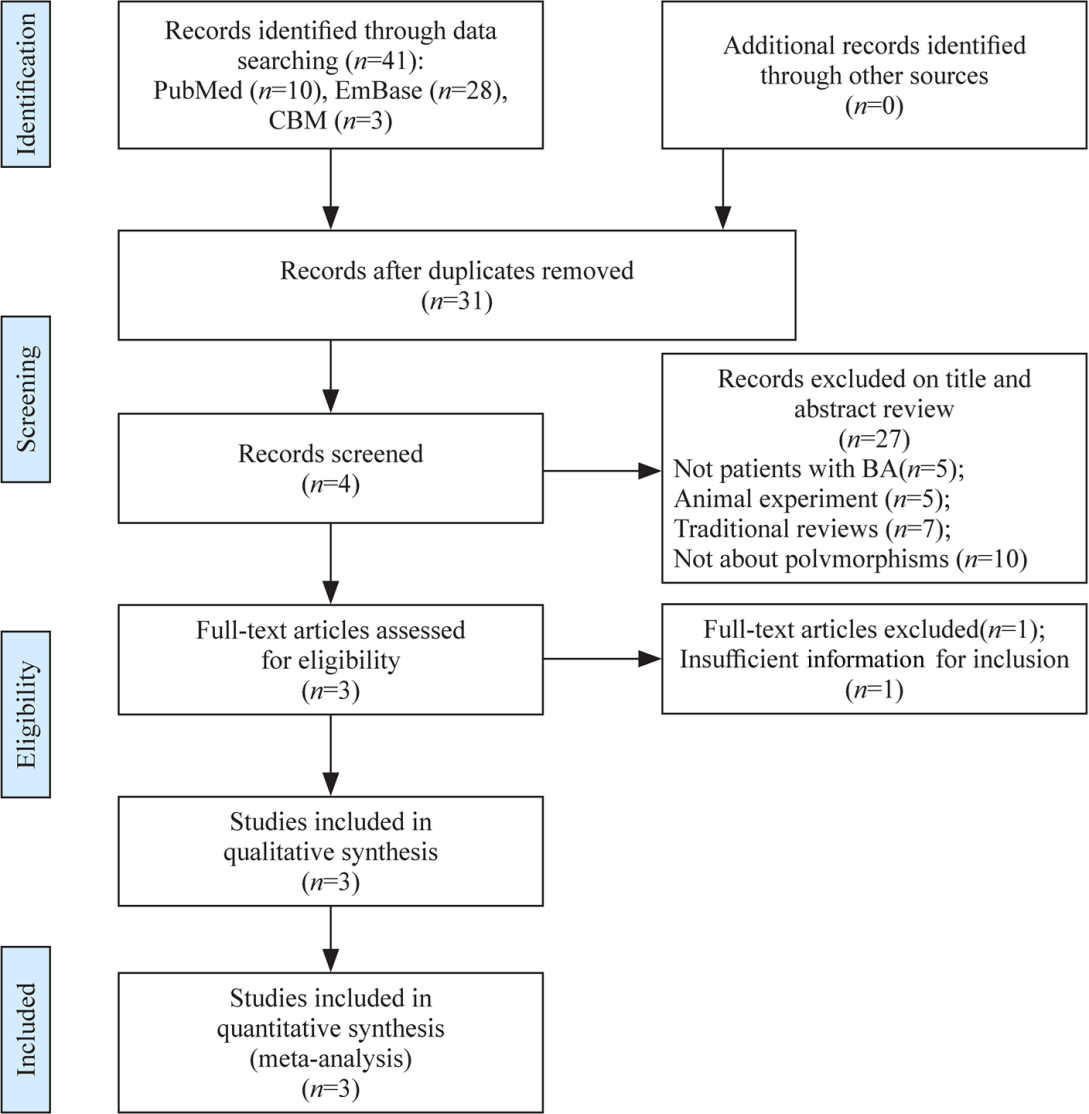
Flow chart of studies considered for inclusion. BA, biliary atresia.

We conducted the search process and reported the findings according to the PRISMA statement^15^ (see figure 1). We identified 41 papers after an initial search. At the first stage, we removed 10 duplicate articles, leaving 31 articles of potential relevance. At the second stage, we excluded 5 papers that did not involve patients with BA, 5 studies on animal experiments, 7 traditional reviews, and 10 studies that were not about polymorphisms. At the final stage, we excluded one paper due to insufficient information for inclusion. Finally, we included three articles^10–12^ for data extraction and meta-analysis.

### Study characteristics

Table 1 shows the main data of the three studies (846 cases and 2821 controls). All of the included studies were conducted in the Chinese population. The genotype distributions of the controls in the three studies^10–12^ were consistent with HWE, but one did not provide exact data.^12^ Of all studies, two^10 12^ involved southern Chinese individuals, and one study^11^ involved northwestern Chinese individuals. All studies^10–12^ used a case–control design. One study^12^ was a case–control cohort study, and another study^11^ was a case–control study but did not report the specific type. All studies used hospital-based controls. The total scores for the three studies ranged from 6 to 9 (see table 1 and online supplemental table S3).

**Table 1:** Characteristics of the studies included in this meta-analysis of VEGFA rs3025039 polymorphism

Study	Population	Area	Race	Sample size		Genotype in cases			Genotype in controls			Minor allele frequency in controls (%)	HWE p value in controls	Type of study	Genotyping method	Quality score
				Cases	Controls	CC	CT	TT	CC	CT	TT					
Lee *et al* 10	Chinese	Southern	Asian	45	160	37	8	0	91	62	7	23.8	>0.05	Hospital-based	TaqMan	6
Liu *et al* 11	Chinese	Northwestern	Asian	311	1205	220	86	5	620	355	50	22.2	0.929	Hospital-based	MassARRAY	6
Liu *et al* 12	Chinese	Southern	Asian	490*	1456*	332	142	16	1002	411	43	17	0.874	Hospital-based	MassARRAY	9

*The number of cases and controls was calculated from the publication.

HWE, Hardy-Weinberg equilibrium; VEGFA, vascular endothelial growth factor A.

### Heterogeneity test

There was a significant between-study heterogeneity in four genetic models of rs3025039 polymorphism (I^2^ range: 66.2%–87.9%, p=0.0003–0.0518; see table 2). Therefore, we used a random-effects model to combine the associations between rs3025039 polymorphism and risk of BA.

**Table 2:** Main results of the meta-analysis

Studies (n)	Comparison model	Test of association			Test of heterogeneity			P value for publication bias	
		OR	95% CI	P value	Q	P value	I^2^ (%)	Begg’s test	Egger’s test
3	C vs T	1.50	0.90 to 2.49	0.121	16.52	0.001	87.9	0.602	0.422
3	CC vs TT	2.02	0.58 to 6.99	0.269	7.49	0.024	73.3		
3	CT+CC vs TT	1.78	0.60 to 5.32	0.299	2.96	0.052	66.2		
3	CC vs CT+TT	1.53	0.89 to 2.63	0.124	5.92	0.001	86.3		

CI, confidence interval; OR, odd ratio.

### Association between rs3025039 and risk of BA

**Figure 2:**
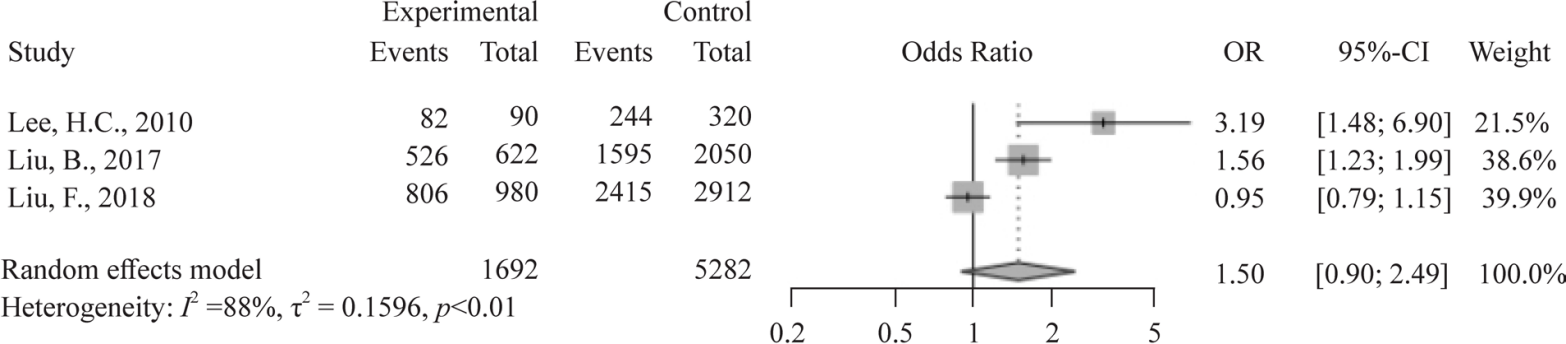
Forest plot of per-allele model of the association between VEGFA rs3025039 polymorphism and biliary atresia. VEGFA, vascular endothelial growth factor A.

Three studies, including 846 cases and 2821 controls, examined the association between rs3025039 and susceptibility to BA. There was no statistically significant association in any of the genetic models (C vs T: OR=1.50, 95% CI 0.90 to 2.49; CC vs TT: OR=2.02, 95% CI 0.58 to 6.99; CT+CC vs TT: OR=1.78, 95% CI 0.60 to 5.32; CC vs CT+TT: OR=1.53, 95% CI 0.89 to 2.63). Table 2 and figure 2 display the distribution of the rs3025039 genotypes and alleles.

### Subgroup analysis

We performed a subgroup analysis by area. Table 3 details the results. Southern Chinese individuals were the subjects in two studies, while one study involved northwestern Chinese individuals. The association between rs3025039 and susceptibility to BA was not significant in southern Chinese patients (C vs T: OR=1.64, 95% CI 0.50 to 5.37; CC vs TT: OR=1.40, 95% CI 0.27 to 7.14; CT+CC vs TT: OR=1.06, 95% CI 0.40 to 2.80; CC vs CT+TT: OR=1.72, 95% CI 0.48 to 6.14). For the northwestern Chinese subgroup, we could not perform a meta-analysis because there was only one study.

**Table 3:** Results of the subgroup analysis

Area	Studies (n)	Comparison model	OR	95% CI
Southern	2	C vs T	1.64	0.50 to 5.37
	2	CC vs TT	1.40	0.27 to 7.14
	2	CT+CC vs TT	1.06	0.40 to 2.80
	2	CC vs CT+TT	1.72	0.48 to 6.14

CI, confidence interval; OR, odd ratio.

### Sensitivity analysis

**Figure 3:**
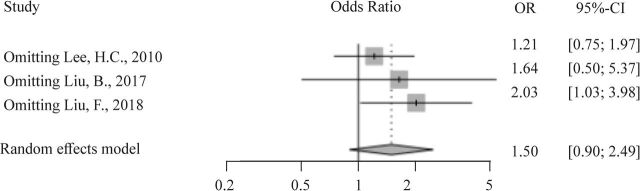
Forest plot of per-allele model for sensitivity analysis.

A sensitivity analysis was performed to evaluate the effects of individual studies on the pooled OR. The pooled OR with 95% CI changed after omitting Liu *et al* 12 (OR=2.03, 95% CI 1.03 to 3.98), suggesting that this study was a source of heterogeneity (I^2^ decreased from 87.9% to 66.9%) (figure 3).

### Publication bias

**Figure 4:**
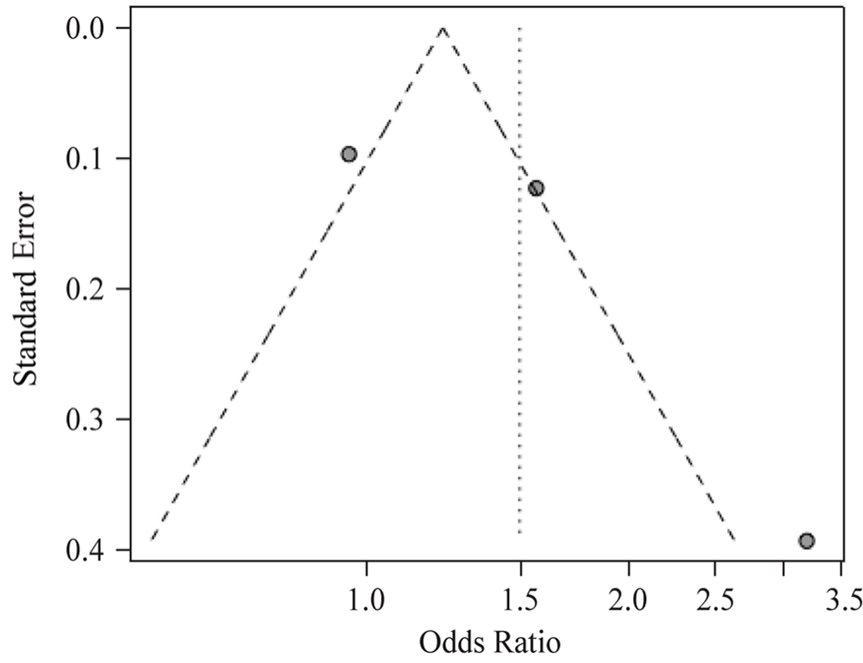
Begg’s funnel plot of publication bias.

Both tests supported the absence of publication bias (p value for Begg’s test=0.602, p value for Egger’s test=0.422; table 2). However, the shape of the funnel plot was asymmetric (figure 4). Because a funnel plot requires at least five studies and because we considered only three studies, there was a possibility that there was a possibility for the funnel plot to show asymmetry. Therefore, we considered only the results of the Begg’s and Egger’s tests.

## Discussion

We conducted a systematic review of the association between *VEGFA* rs3025039 polymorphism and susceptibility to BA. Lee *et al* 10 were the first to identify *VEGFA* as a susceptibility locus for BA in Chinese patients. This result has biological plausibility; *VEGFA* is a mediator of the pathogenesis of BA. As a proinflammatory cytokine, *VEGFA* participates in various processes including cell proliferation, cell migration, and apoptosis, all of which occur in cell-mediated immune-inflammatory diseases, such as BA.^5 6 8^ There are several independent verifications of this association, but the conclusions remain controversial because the results of different studies in China were conflicting.^11 12^

We found a difference between cases and controls in comparisons of all genotypes of rs3025039, suggesting that rs3025039 polymorphism may not correlate with susceptibility to BA. A subgroup analysis by area further suggested no significant association between rs3025039 and susceptibility to BA. Given that only one study included the subgroup of northwestern Chinese, our study did not demonstrate a solid correlation between rs3025039 and BA in northwestern Chinese patients.

The absence of significant findings may stem from the fact that we included only three original studies in this study. In addition, as the sample size increased, the association between rs3025039 and susceptibility to BA decreased in the three studies. We observed statistical significance in only two studies with small numbers of participants. Therefore, the statistical power was relatively low, suggesting that the association might be spurious. For the first reason, more original studies regarding the association between rs3025039 and susceptibility to BA are necessary to generate accurate results. However, for the second reason, the effect may be relatively small or not at all, and explicit exploration of this association may result in an unnecessary study. Therefore, it may be more cost-effective to devote more resources to explore other potential biomarkers.

Several limitations need to be acknowledged when considering the results of our study. First, the quantity and sample size of the included studies were insufficient to obtain high power for making a confirmatory conclusion, even though we undertook a comprehensive literature search. Second, all study subjects came from China only; therefore, we could not avoid potential selection bias. Third, residual confounders were possible because BA is a multifactorial and complicated disease, involving gene–environment and gene–gene interactions. We could not detect these effects due to limited information. Fourth, there was a lack of sufficient data; therefore, we did not conduct a subgroup analysis of familial and other BA types. Finally, there was significant heterogeneity that might distort the results. Several aspects may cause heterogeneity, including differences in experimental methods across studies. Therefore, readers should interpret our results with caution.

Despite the limitations, we believe that the present study provides useful evidence regarding the role of *VEGFA* rs3025039 polymorphism in BA. The sample size of each study included in this study was insufficient to make a definite conclusion regarding the association between rs3025039 polymorphism and susceptibility to BA; however, the pooled OR calculated from the three studies significantly increased the statistical power. It is worth noting that the sample size increased from 205 subjects in the first study to 3667 subjects in our study. Reaching a relative sufficient statistical power is crucial in GAS. Furthermore, no significant publication bias was found in this study and the result was relatively stable in the subgroup analysis.

In conclusion, *VEGFA* rs3025039 polymorphism might not be associated with an elevated risk of BA in Chinese population. Obtaining a definitive conclusion regarding this association may entail additional research. Future studies are recommended to identify other possible genetic markers.

## Data Availability

Data are available in a public, open access repository. As a meta-analysis, all of the data in this study can be found in table 1 and were extracted from the original studies.
