# Supplementary material for: VEGFA rs3025039 and biliary atresia susceptibility in Chinese population: a systematic review and meta-analysis
Source: World J Pediatr Surg. 2022 Jan 6;5(1):e000344. doi: 10.1136/wjps-2021-000344 (PMC9717001; doi:10.1136/wjps-2021-000344)
Supplement: Supplementary data [file wjps-2021-000344supp001.pdf]

PubMed（1900/00/00-2020/08/18）

((((Biliary atresia[MeSH Terms]) OR ((((((((((Biliary atresia[Title/Abstract]) OR (Atresia, Biliary[Title/Abstract])) OR (Intrahepatic Biliary Atresia[Title/Abstract])) OR (Atresia, Intrahepatic Biliary[Title/Abstract])) OR (Biliary Atresia, Intrahepatic[Title/Abstract])) OR (Biliary Atresia, Extrahepatic[Title/Abstract])) OR (Atresia, Extrahepatic Biliary[Title/Abstract])) OR (Extrahepatic Biliary Atresia[Title/Abstract])) OR (Idiopathic Extrahepatic Biliary Atresia[Title/Abstract])) OR (Familial Extrahepatic Biliary Atresia[Title/Abstract])))) AND ((((((((((((Vascular Endothelial Growth Factor-A[Title/Abstract]) OR (VEGF-A[Title/Abstract])) OR (VEGFA[Title/Abstract])) OR (Vasculotropin[Title/Abstract])) OR (VEGF[Title/Abstract])) OR (Vascular Endothelial Growth Factor[Title/Abstract])) OR (Vascular Permeability Factor[Title/Abstract])) OR (Permeability Factor, Vascular[Title/Abstract])) OR (Glioma-Derived Vascular Endothelial Cell Growth Factor[Title/Abstract])) OR (Glioma Derived Vascular Endothelial Cell Growth Factor[Title/Abstract])) OR (GD-VEGF[Title/Abstract])) OR ((Vascular Endothelial Growth Factor A[MeSH Terms]) OR (Vascular Endothelial Growth Factors[MeSH Terms])))) AND (("1900/01/01"[Date - Publication] : "2020/08/18"[Date - Publication]))

Embase（1900/00/00-2020/08/18）

No.	Query	Results	Date
#30	#12 AND #27 AND [1-1-1900]/sd NOT [18-8-2020]/sd	28	20-Aug-20
#29	#12 AND #27 AND [1-1-1900]/sd NOT [19-8-2020]/sd	28	20-Aug-20
#28	#12 AND #27	28	20-Aug-20
#27	#13 OR #14 OR #15 OR #16 OR #17 OR #18 OR #19 OR #20 OR #21 OR #22 OR #23 OR #24 OR #25 OR #26	151830	20-Aug-20
#26	vascular endothelial growth factors':ti,ab	1250	20-Aug-20
#25	vascular endothelial growth factor a':ti,ab	4416	20-Aug-20
#24	gd-vegf':ti,ab	2	20-Aug-20
#23	glioma derived vascular endothelial cell growth factor':ti,ab	1	20-Aug-20
#22	glioma-derived vascular endothelial cell growth factor':ti,ab	1	20-Aug-20
#21	permeability factor, vascular':ti,ab	123	20-Aug-20
#20	vascular permeability factor':ti,ab	687	20-Aug-20
#19	vascular endothelial growth factor':ti,ab	74734	20-Aug-20
#18	vegf':ti,ab	98548	20-Aug-20
#17	vasculotropin':ti,ab	13	20-Aug-20
#16	vegfa':ti,ab	12762	20-Aug-20
#15	vegf-a':ti,ab	9885	20-Aug-20

#14	vascular endothelial growth factor-a':ti,ab	4416	20-Aug-20
#13	vasculotropin'/exp	109406	20-Aug-20
#12	#1 OR #2 OR #3 OR #4 OR #5 OR #6 OR #7 OR #8 OR #9 OR #10 OR #11	8458	20-Aug-20
#11	familial extrahepatic biliary atresia':ti,ab	5	20-Aug-20
#10	idiopathic extrahepatic biliary atresia':ti,ab	1	20-Aug-20
#9	extrahepatic biliary atresia':ti,ab	615	20-Aug-20
#8	atresia, extrahepatic biliary':ti,ab	5	20-Aug-20
#7	biliary atresia, extrahepatic':ti,ab	1	20-Aug-20
#6	biliary atresia, intrahepatic':ti,ab	7	20-Aug-20
#5	atresia, intrahepatic biliary':ti,ab	0	20-Aug-20
#4	intrahepatic biliary atresia':ti,ab	34	20-Aug-20
#3	atresia, biliary':ti,ab	27	20-Aug-20
#2	biliary atresia':ti,ab	6581	20-Aug-20
#1	bile duct atresia'/exp	7634	20-Aug-20

CBM（1900-2020）

("胆道闭锁"[摘要]) AND (("血管内皮生长因子 A"[摘要] OR "血管内皮生长因子"[摘要] OR "VEGFA"[摘要] OR "VEGF"[摘要] OR "Vascular Endothelial Growth Factor A"[摘要] OR "Vascular Endothelial Growth Factors"[摘要] OR "Vascular Endothelial Growth Factor"[摘要]) AND 1900-2020[日期])
